# Construct validity and reliability of the Malay version of the Fagerström test for nicotine dependence (FTND): A confirmatory factor analysis

**DOI:** 10.18332/tid/159624

**Published:** 2023-03-08

**Authors:** Kuang Hock Lim, Yah Xin Yun, Yoon Ling Cheong, Norharlina Sulaiman, Mas Eliana Mahadzir, Jia Hui Lim, Mohd Hazilas Mat Hashim, Hui Li Lim

**Affiliations:** 1 Institute for Medical Research, National Institutes of Health, Kuala Lumpur, Malaysia; 2 Selangor Pharmacy Enforcement Branch, Selangor State Health Department, Selangor, Malaysia; 3 Pharmacy Department, Hospital Sungai Buloh, Sungai Buloh, Malaysia; 4 Pharmacy Department, Shah Alam Hospital, Shah Alam, Malaysia; 5 Pharmacy Department, Putrajaya Hospital, Putrajaya, Malaysia; 6 Clinical Research Centre, Hospital Sultan Ismail, Johor Bahru, Malaysia

**Keywords:** FTND, nicotine addiction, confirmatory factor analysis, daily smokers

## Abstract

**INTRODUCTION:**

The Fagerström test for nicotine dependence (FTND) was forward-backwards translated into the Malay language (FTND-M) and administered to 152 daily smokers who sought treatment for smoking cessation in government health clinics in Selangor state, Malaysia.

**METHODS:**

Using confirmatory factor analysis (CFA), four measurement models with the best relative fit were compared, one uni-dimensional model, and three different two-domain (morning and daytime smoking) models.

**RESULTS:**

The findings indicate that the best model of the FTND-M was a two-domain model, wherein domain one represented morning smoking (time to first cigarette of the day, smoking more in the morning, and which cigarette would you hate to give up) and domain two represented daytime smoking (cigarettes per day, difficulty refraining from smoking, and smoking when ill) which showed good model fit [χ^2^/df=1.932, goodness of fit (GFI) of 0.967, comparative fix index (CFI) of 0.945, incremental fit index (IFI) of 0.98, Tucker-Lewis index (TLI) of 0.95 and a real mean square end of approximation (RMSEA) of 0.079, and substantial reliability >0.70].

**CONCLUSIONS:**

This study indicates that the FTND-M can be used to assess these two dimensions of nicotine addiction among daily smokers in a clinical setting.

## INTRODUCTION

Cigarette smoking is the root cause of numerous public health problems that are the leading causes of death globally^1,2^. Addiction to nicotine is one of the main barriers to smoking cessation among smokers^3^. An accurate cigarette addiction assessment tool is required for screening purposes and use in research. The Fagerström test for nicotine dependence (FTND), which originated from the Fagerström Tolerance Questionnaire^4^ and later was modified by Heatherton et al.^5^, is one of the most widely used and accepted instruments for determining and quantifying nicotine addiction in the past few decades^6-8^. However, there is still disagreement on the reliability of the FTND. For instance, Heatherton et al.^5^, Payne et al.^9^, and Haddock et al.^10^, reported relatively low Cronbach alpha values of the FTND, 0.61, 0.56, and 0.67 respectively, far short of the minimum of 0.80 recommended by Nunnally and Bernstein^11^. In addition, various language-translated versions of the FTND reportedly consist of two dimensions^12-17^, contrary to the uni-dimensionality of the original FTND^5^. Researchers who reported the bi-dimensionality of FTND suggested the first domain, which is characterized by morning smoking-related questions, which is thought to measure how urgently nicotine levels need to be brought up to a certain threshold following nighttime abstinence, whilst the second domain has been interpreted as a measure of the persistence with which nicotine levels are maintained at a certain threshold during waking hours.

However, researchers report different items in both domains of FTND, with Radzius et al.^12^, Richardson and Ratner^14^, and Uysal et al.^15^ reporting items 1, 2 and 3: time to the first cigarette of the day, smoke more in the morning, and which cigarette would you hate to give up (Table 1), in the first domain; while in the second domain are the remaining items 4, 5 and 6: cigarettes per day, difficulty refraining from smoking, and smoking when ill (Table 1). However, de Meneses-Gaya et al.^16^ and Huang et al.^17^ reported only two items (smoke more in morning and which cigarette would you hate to give up) in the first domain while the remaining four items were in the second domain. We postulate that social and cultural differences and understanding of the items asked, are among the factors that contribute to the differences in the items in the first and second domain. While in Malaysia, a study conducted by Yee et al.^18^ on 107 male smokers at the University Medical Center and using the EFA Technique and Obilimin rotation, reported two domains with different items from the previous study (the first domain consists of: time to first cigarette of the day, which cigarette do you most hate to give up, and cigarettes per day; while the second domain consists of: difficult to refrain from smoking in forbidden places, ‘increase smoking in the morning and smoking during illness). However, EFA is typically used during the early stages of scale development, when the researcher has little familiarity with the underlying component structure. Even if a researcher selects the ultimate solution by considering one or more qualitative or quantitative criteria, it is probably better to be regarded as a hypothetical measuring model that has not been fully assessed. Contrary to exploratory factor analysis (EFA), confirmatory factor analysis (CFA) is a hypothesis-testing technique that statistically assesses the reliability of a predetermined measurement model. The researcher must specify each element of the model *a priori*, before conducting a CFA^19^. Since the validity and domains of the FTND have already been established, it is more appropriate to conduct a CFA to determine the construct validity and reliability of FTND-M. However, no such study has been carried out in Malaysia. Therefore, this investigation aims to address this gap by evaluating the construct validity and reliability of the FTND-M, among Malaysian adult daily smokers.

**Table 1:** Items in FTND and their Malay translation

*No*	*Item*
1	How soon after you wake up do you smoke your first cigarette? *Berapa cepatkah anda menghisap rokok anda yang pertama selepas bangun dari tidur?*
2	Do you smoke more frequently during the first hours after waking than during the rest of the day? *Adakah anda lebih banyak merokok dalam beberapa jam pertama selepas bangun tidur berbanding dengan waktuwaktu lain sepanjang hari?*
3	Which cigarette would you hate most to give up? *Menghisap rokok yang manakah yang paling sukar untuk anda tinggal?*
4	How many cigarettes per day do you smoke? *Berapa batang rokok yang anda hisap dalam sehari?*
5	Do you find it difficult to refrain from smoking in places where it is forbidden (for example: in the movie theater, in the library, in church)? *Adakah anda menghadapi kesukaran menahan diri dari merokok di tempat-tempat yang dilarang merokok, contohnya di rumah ibadat, di perpustakaan,di panggung wayang dan sebagainya?*
6	Do you smoke if you are so ill that you are in bed most of the day? *Adakah anda merokok walaupun anda sakit dan berada dalam keadaan di mana anda terpaksa berbaring di atas katil sepanjang hari?*

FTND: Fagerström test for nicotine dependence.

## METHODS

We extracted data from a study investigating the prevalence and risk factors of quitting among daily smokers seeking treatment at a government quit smoking clinic in Selangor from June 2017 to December 2019. The smoking cessation treatment included advice on behavioral changes and provision of pharmaceutical therapy, including nicotine gum, varenicline etc. A representative sample of daily smokers was selected using two-stage sampling. The first stage was the selection of government smoking cessation clinics in Selangor. In the second stage, systematic random sampling was used to choose respondents in the selected clinics. Assuming 10% prevalence of smoking cessation and 5% precision, a minimum sample size of 139 was needed. After incorporating an additional 10% to account for potential non-response, the sample size was expanded to 153.

The intercept method was used for enlisting participants in this study. Every third person seeking smoking cessation treatment encountered at the clinic was invited to take part in the study. If the respondent failed to meet the inclusion criteria (Malaysian aged ≥21 years and able to understand Malay or English language) or declined to take part, the next person was approached. Members of the research team described the objectives and methodology of the study, and the conditions of anonymity, protection and confidentiality of the data. After receiving the above explanation, respondents signed an informed consent form and a trained pharmacist then proceeded to interview the respondents. The study protocol was vetted and approved by the Malaysian Research and Ethics Committee of the Ministry of Health Malaysia.

Development of the FTND-M was conducted as follows. The FTND was first translated into Malay by a content expert (a public health professional) and an English language graduate teacher with at least five years of teaching experience. The Malay version was then translated back into English by two additional subject and language experts. After reviewing both versions with the research team, the translation and research teams unanimously agreed on the final questionnaire. The team made several language and cultural context adjustments after comparing the original FTND with the Malay version in order to finalize the translation. The final version of the FTND-M was thus obtained and employed in this investigation. In addition to the FTND-M, the questionnaire also contained items on sociodemographic variables, smoking status, age started smoking, number of cigarettes smoked per day, and type of cigarette. Daily smokers were defined as those who smoked a cigarette at least once a day. We excluded individuals who used other types of tobacco products from the analysis.

Four different FTND-M models were tested using CFA. Model 1: Uni-dimensional factor model of the FTND-M with items 1–6 (Table 1) loading into one domain; Model 2: model composed of two correlated factors: Items 1–3 (Table 1) loading on a morning smoking domain and Items 4–6 (Table 1) loading on a daytime smoking domain based on the findings of Radzius et al.^12^, Richardson et al.^14^, Payne et al.^9^, and Ulysal et al.^15^. Model 3 was based on the findings of Yee et al.^18^ among Malaysian male smokers: Items 1, 3, 4 in the morning smoking domain; and Items 2, 5 and 6 (Table 1) in the daytime smoking domain. Model 4 was based on the studies by de Maneses-Gaya et al.^16^ and Huang et al.^17^: Items 2 and 3 in the morning smoking domain; and Items 1, 4, 5 and 6 (Table 1) in the daytime smoking domain. A model-testing technique recommended by Bollen^20^ was used to assess the four models. CFA model fit was evaluated using multiple fit indices, namely relative chi-squared, goodness of fit index (GFI), adjusted goodness of fit index (AGFI), comparative fit index (CFI), root mean square error of approximation (RMSEA), incremental fit index (IFI), and Tucker-Lewis index (TLI). The reliability of the FTND-M was only carried out on the model that fit and is assessed by construct reliability. AMOS software was used to perform the analysis. All statistical analyses were performed at the 95% significance level.

## RESULTS

Of the 153 respondents, data from 152 (99.4%) respondents were finally included in the study. The majority of respondents were male (n=142; 93.4%). The respondents were aged 44.4 years on average (SD=13.05). They were mostly married (76.3%). Almost one-third (35.5%) said they had completed tertiary education (Table 2). The average daily tobacco consumption was 17.31 cigarettes (SD=5.54). The average age at smoking initiation and daily smoking was 16.78 (SD=5.45) and 19.26 years (SD=6.29), respectively. Approximately four in ten (40.5%) started smoking daily before the age of 18 years. The mean FTND score was 4.16 (SD=2.67) (range: 0–10).

**Table 2:** Sociodemographic characteristics of daily smokers attending government smoking cessation clinics in Selangor related to the Malay FTND Score (N=152)

*Characteristics*	*n*	*%*	*FTND score Mean (SD)*
**Gender**			
Male	142	93.4	4.07 (2.66)
Female	10	6.6	5.40 (2.67)
**Locality**			
Urban	89	58.6	4.42 (2.28)
Rural	63	41.4	3.78 (3.12)
**Age** (years)			
18–29	21	13.8	3.48 (2.87)
30–39	37	24.3	5.05 (2.65)
40–49	36	23.7	4.06 (2.53)
50–59	33	21.7	3.76 (2.72)
≥60	25	16.4	4.08 (2.55)
**Ethnicity**			
Malay	101	66.4	4.16 (2.94)
Chinese	26	17.1	4.23 (2.20)
Indian	21	13.8	4.00 (2.14)
Other	4	2.6	4.50 (0.58)
**Education level**			
Primary–Secondary	98	64.5	4.31 (2.31)
Tertiary	54	35.5	3.89 (2.79)
**Marital status**			
Single/divorced	36	23.7	4.94 (2.80)
Married	116	76.3	3.91 (2.60)

SD: standard deviation.

As seen in Table 3, approximately a third of smokers (29.6%) reported smoking within five minutes of waking up in the morning. Regarding smoking habits, 40.8% had difficulty refraining from smoking in a place where it is forbidden (Item 2). More than half of the smokers said that the first cigarette in the morning is the most difficult to give up (Item 6) and more than 4 in 10 respondents smoked more frequently in the morning. The highest mean score was for Item 1: the first cigarette in the morning (mean=2.40; SD=1.17).

**Table 3:** Fagerström test for nicotine dependence scoring, response and item mean scores of the Malay FTND (N=152)

*No*	*Item*	*Response categories*	*Score*	*n (%)*	*Mean score (SD)*
1	How soon after you wake up do you smoke your first cigarette?	Within 5 minutes	3	45 (29.6)	2.40 (1.17)
		6–30 minutes	2	41 (27.0)	
		31–60 minutes	1	26 (17.1)	
		After 60 minutes	0	40 (26.3)	
2	Do you find it difficult to refrain from smoking in places where it is forbidden, like cinema, church etc.?	Yes	1	62 (40.8)	0.41 (0.49)
		No	0	90 (59.8)	
3	Which cigarette do you hate most to give up?	The first one in the morning	1	86 (56.6)	0.57 (0.50)
		All others	0	66 (43.4)	
4	How many cigarettes/day do you smoke?	≤10	0	59 (38.8)	0.91 (0.95)
		11–20	1	63 (41.4)	
		21–30	2	14 (9.2)	
		≥31	3	16 (10.5)	
5	Do you smoke more frequently during the first hour after waking than during the rest of the day?	Yes	1	46 (30.3)	0.30 (0.46)
		No	0	106 (69.7)	
6	Do you smoke if you are so ill that you are in bed most of the day?	Yes	1	56 (36.8)	0.37 (0.48)
		No	0	96 (63.2)	

SD: standard deviation.

Table 4 presents the results of our primary analyses which indicated that only Models 2 and 4 had an adequate model fit, with Model 2 having higher and more substantial construct reliability (>0.70) for both domains in the model, whereas Model 4 had lower construct reliability of 0.486 and 0.498, respectively for both domains. The uni-dimensional model and the two domains model did not display satisfactory model fit in CFA analysis.

**Table 4:** Comparison of the fit indices of four confirmatory factor analysis models of the Malay FTND (FTND-M)

*Fit indices*	*Model 1*	*Model 2*	*Model 3*	*Model 4*
Model χ^2^ (df)	34.58 (9)	15.47 (8)	27.10 (8)	15.78 (8)
p	<0.001	0.051	0.001	0.046
χ^2^/df	3.843	1.932	3.388	1.973
AGFI	0.725	0.914	0.856	0.880
GFI	0.82	0.967	0.945	0.602
CFI	0.827	0.947	0.871	0.947
IFI	0.833	0.95	0.876	0.950
TLI	0.711	0.95	0.757	0.901
RMSEA	0.137	0.079	0.126	0.08
Construct reliability		Domain 1: 0.752 Domain 2: 0.743		Domain 1: 0.486 Domain 2: 0.498

Model 1: Uni-dimensional model; Items 1–6 (Table 1). Model 2: Two domains model; Domain one: Items 1–3; Domain two: 4–6 (Table 1). Model 3: Two domains model; Domain one: Items 1, 3 and 4; Domain two: 2, 5 and 6 (Table 1). Model 4: Two domains model; Domain one: Items: 2 and 3; Domain two: 1, 4, 5 and 6 (Table 1).

## DISCUSSION

In this study, we used CFA to compare several models of the FTND using statistical software that was specifically designed to facilitate the modelling of ordered categorical data using small samples. The findings support earlier exploratory factor studies that suggested the FTND’s component structure was not one-dimensional but rather comprised two associated factors^12-16^. The first element, which is characterized by questions about morning smoking, Items 1, 2 and 3 (Table 1), is thought to measure how urgently nicotine levels need to be brought back to a certain threshold following nighttime abstinence. The second component appears to evaluate smoking habits during the day and is determined by Items 4, 5 and 6 (Table 1). It has been interpreted as a gauge of how persistently nicotine levels are elevated. As with previously published EFAs and CFA^12-16^, the current study appears to strongly support the robustness of this multi-dimensional structure in that the assessments of relative fit (i.e. which of the nested models fit best), absolute fit (which models passed the chi-squared test), and approximate fit (RMSEA, SRMR, TLI, and CFI) all indicated that a two-factor solution (Model 2) provided the best fit to the data. Furthermore, in addition to this study, the same two-factor solution consistently emerged in several previously published EFAs and CFAs, despite there being substantial differences in the demographics of the sampled populations. For example, Payne et al.^9^ examined veterans, their spouses and hospital employees enrolled in a smoking cessation clinic (respondent mean age 49 years). Both Haddock et al.^10^ and Radzius et al.^12^ used patient scores from the National Institute on Drug Abuse Intramural Research Program of the National Institute of Health to assess candidates entering the US Air Force Basic Military Training program (mean age 20 years) (83% had a clinical diagnosis for substance dependence other than nicotine; mean age of 36 years). In addition, Uysal et al.^15^ reported similar results in their investigation among smokers in Turkey. However, the items in the first and second domains were different from de Maneses-Gaya et al.^16^ and Hu et al.^17^, who reported that domain 1 consisted of Items 2 and 3 (Table 1), and the second domain included Items 1, 4, 5 and 6 (Table 1). These differences may be due to the differing characteristics of respondents between studies.

The substantial reliability (>0.70) of the two domains in this study is similar to the 0.80 reported in a study by Richardson for domain 1 and 0.73 for domain 214, and to Huang et al.17 who reported construct reliability of 0.74 in their study. Overall, our study showed that the six items of the FTND-M had satisfactory validity that confirms its structure as that of a two-domain model. This study contributes to understanding the utility of the FTND in different sociocultural settings.

### Limitations

Our study has some limitations. First, the results of this study, are generalizable to people who visit smoking cessation clinics seeking treatment for daily smoking and cannot be applied to the general community. Second, because of the challenging recruitment process and the low prevalence of female smokers in Malaysia (just 6.6% of the study sample was female), it is necessary to conduct more research on female smokers and to develop the concept of cigarette dependence in order to address particular characteristics in cultural contexts. Thirdly, test-retest data reliability was not carried out in this study, which, if it had been done, would have added more value to this study. However, the validity and reliability of the two domains of the Malaysian FTND found in this study will enable the use of the instruments by an allied health worker and aid medical practitioners in assessing the patient’s nicotine dependence and identifying those who should be referred for cessation therapy.

## CONCLUSIONS

The Malay version of FTND displayed good psychometric performance among the daily smokers seeking treatment in smoking cessation clinics, and allied health workers can use this instrument in other clinic settings in Malaysia that offer smoking cessation services

## Data Availability

The data supporting this research are available from the authors on reasonable request.
